# A Tailored Postpartum eHealth Physical Activity Intervention for Individuals at High Risk of Postpartum Depression—the POstpartum Wellness Study (POW): Protocol and Data Overview for a Randomized Controlled Trial

**DOI:** 10.2196/56882

**Published:** 2024-10-29

**Authors:** Maya Ramsey, Nina Oberman, Charles P Quesenberry Jr, Elaine Kurtovich, Lizeth Gomez Chavez, Aaloni Chess, Susan Denise Brown, Cheryl L Albright, Mibhali Bhalala, Sylvia E Badon, Lyndsay A Avalos

**Affiliations:** 1 Division of Research, Kaiser Permanente Northern California Pleasanton, CA United States; 2 School of Medicine University of California, Davis Sacramento, CA United States; 3 University of Hawaii at Manoa Honolulu, HI United States; 4 Redwood City Medical Center, Kaiser Permanente Northern California Redwood City, CA United States

**Keywords:** postpartum depression, depression, eHealth, online workout videos, exercise videos, physical activity, wellness, health promotion, digital interventions

## Abstract

**Background:**

Postpartum depression (PPD) is associated with significant health consequences for the parent and child. Current recommendations for PPD prevention require intense health care system resources. Evidence-based interventions for PPD prevention that do not further burden the health care system are needed. Evidence suggests that physical activity (PA) can generally reduce depressive symptoms. Technology-based interventions may help decrease common barriers to PA.

**Objective:**

This study aims to report the protocol and provide a data overview of the POstpartum Wellness study (POW)—an effectiveness trial evaluating whether an eHealth PA intervention tailored for postpartum individuals increased PA and decreased depressive symptoms among individuals at high PPD risk.

**Methods:**

This remote parallel-group randomized controlled trial included postpartum individuals with a history of depression or at least moderate current depressive symptoms not meeting the PPD diagnostic threshold and with low PA levels from an integrated health care delivery system. Participants were randomized to an eHealth PA intervention or usual care. The intervention group received access to a library of web-based workout videos designed for postpartum individuals, which included interaction with their infants. At baseline and follow-up (3 and 6 months), PA was measured using questionnaires and a wrist-worn accelerometer. Depressive symptoms were measured using the Patient Health Questionnaire-8 (PHQ-8). Data were collected to assess exploratory outcomes of sleep, perceived stress, anxiety, parent-infant bonding, and infant development.

**Results:**

The study was funded in January 2020. Participants were enrolled via REDCap (Research Electronic Data Capture) or telephonically between November 2020 and September 2022; data collection ended in April 2023. Randomized participants (N=99) were 4 months post partum at baseline with moderately severe depressive symptoms (mean PHQ-8 score 12.6, SD 2.2). Intervention (n=50) and usual care (n=49) groups had similar sociodemographic characteristics, months post partum, baseline depressive symptoms, number of children at home, and prepregnancy PA levels. Retention in assessments was ≥66% for questionnaires and ≥48% for accelerometry, with modest differences by group. At 3-month follow-up, 73 of 99 (74%) participants (intervention: 35/50, 70%; usual care: 38/49, 78%) completed questionnaires; 53 of 99 (54%) wore the accelerometer for 7 days (27 of 50 (54%) intervention, 26 of 49 (53%) usual care). At 6-month follow-up, 66 of 99 (67%) participants (30 of 50 (60%) intervention, 36 of 49 (73%) usual care) completed questionnaires and 43 of 99 (43%) wore the accelerometer for 7 days (21 of 50 (42%) intervention, 22 of 49 (45%) usual care). Data analysis is completed, and a manuscript with these findings is currently under review for publication.

**Conclusions:**

The POW trial evaluates the effectiveness of an eHealth PA intervention for improving depressive symptoms and increasing PA among postpartum individuals at high PPD risk. Results have implications for the design and delivery of behavioral interventions among vulnerable patients.

**Trial Registration:**

ClinicalTrials.gov NCT04414696; https://clinicaltrials.gov/ct2/show/NCT04414696

**International Registered Report Identifier (IRRID):**

DERR1-10.2196/56882

## Introduction

Postpartum depression (PPD) is a debilitating and costly condition that affects over 22% of birthing parents and is associated with significant health consequences for them [1-5] and their children [6-12]. In 2019, the US Preventive Services Task Force issued a recommendation stating that postpartum women at increased risk of PPD (ie, women with a history of depression prior to pregnancy or at least moderate postpartum depressive symptoms that do not meet the diagnostic threshold for PPD) [13,14] should receive counseling interventions [15]. We have previously [16] highlighted the significant pressure this will place on the demands of health care systems already struggling with a shortage of mental health care providers. Thus, effective, evidence-based interventions to prevent PPD among those at risk—that can be easily integrated into health care systems, yet do not involve intensive health care system resources—are urgently needed.

Strong evidence in general populations suggests physical activity (PA) can reduce depression risk by half [17-21]. While national guidelines and professional organizations, including the American College of Obstetricians and Gynecologists, recommend at least 150 minutes per week of moderate to vigorous intensity PA for postpartum women [22,23], 70% of postpartum women do not meet these guidelines [24-29]. Common barriers to participating in postpartum PA include parental responsibilities and childcare, limited time, and limited availability and awareness of existing PA resources for postpartum individuals [30]. Technology-based (eHealth) interventions are a promising approach since they can address these common barriers and have been shown to effectively increase PA in the general population [31,32]. However, there are few eHealth PA interventions tailored specifically for postpartum women, and it is unclear if such interventions are effective in reducing PPD risk.

The POstpartum Wellness study (POW) is an effectiveness randomized controlled trial (RCT) to evaluate whether an eHealth PA intervention tailored for postpartum individuals was effective at increasing PA and decreasing depressive symptoms among postpartum individuals at increased risk of PPD and with low PA. Here, we provide a detailed overview of the trial protocol following the CONSORT (Consolidated Standards of Reporting Trials) guidelines, and present baseline data and retention in trial follow-up assessments.

## Methods

### Study Setting

This trial was conducted in Kaiser Permanente Northern California (KPNC), an integrated health care delivery system that provides care for over 4.6 million members (over 66,000 pregnant and postpartum individuals annually). KPNC health plan members are covered by employer-sponsored insurance plans, the California Insurance Exchange, Medicare, and Medicaid. Coverage is provided for approximately 47% of the Northern California population and is similar demographically, ethnically, and socioeconomically to the underlying population except with respect to income, where members underrepresent the very poor and the very wealthy [33,34]. As part of standard perinatal care, women are screened for PPD using the validated Patient Health Questionnaire-9 (PHQ-9) screening tool [35,36] at their 4- to 6-week postpartum visit [37,38]. Additionally, postpartum parents are screened for PPD using the Patient Health Questionnaire-2 (PHQ-2) [39] at each well-baby visit. All screening scores are captured in the KPNC’s comprehensive electronic health records (EHR).

### Study Design Overview

POW is a 2-arm parallel RCT comparing an eHealth PA intervention tailored for postpartum women to usual postpartum care in women at high risk of PPD with low PA levels. Potential participants were identified via PPD screening scores captured in KPNC’s EHR databases or clinician referral. Enrolled participants completed assessments at baseline before randomization, and at follow-ups at 3 months and 6 months post randomization. Primary outcomes were depressive symptoms and device-based PA at 3-month follow-up. Secondary outcomes were depressive symptoms and device-based PA at 6-month follow-up and self-reported PA (3-month follow-up and 6-month follow-up). Additional outcomes ascertained included self-reported sleep, anxiety, stress, parent-infant bonding, and parent-reported infant development. See Table 1 for a list of data collected at each time point. Recruitment began on November 19, 2020. The trial protocol was modified 3 times to improve trial implementation (Modification 1 in January 2021, Modification 2 in July 2021, and Modification 3 in February 2022), as described below and in Table 2.

**Table 1:** Data collection and time points.

Measures		Instrument	Recruitment screener	Baseline	3 months post baseline	6 months post baseline	12 months post partum
**Primary and Secondary Outcomes^a^**							
	Depressive symptoms	Patient Health Questionnaire-8 (PHQ-8) [40]	X		X	X	
	Moderate/vigorous intensity physical activity	ActiGraph GT3X+ (7 days); Pregnancy Physical Activity Questionnaire (PPAQ) [41]		X	X	X	
**Additional Outcomes**							
	Perceived stress	Perceived Stress Scale (PSS-10) [42]		X	X	X	
	Anxiety symptoms	Generalized Anxiety Disorder (GAD-7) [43]		X	X	X	
	Sleep quality and duration	Pittsburg Sleep Quality Index (PSQI) [44]		X	X	X	
	Mother and infant bonding	Mother-to-Infant Bonding Scale (MIBS) [45]		X	X		
	Infant development at 12 months	Ages and Stages Questionnaire (ASQ-3) [46]					X
	Participant satisfaction				X		
**Potential Mediators for Physical Activity (PA)**							
	Self-efficacy	Modified self-efficacy to overcome barriers to PA Scale [47]		X	X		
	Perceived barriers	Modified barriers to being physically active scale [47]		X	X		
**Potential Effect Modifiers**							
	Physical activity before pregnancy	Stanford Leisure-Time Activity Categorical Item (L-Cat) [48], modified to reflect year before pregnancy		X			
	Physical activity during pregnancy	Stanford L-Cat, modified to reflect pregnancy		X			
**Covariates and Confounders**							
	Demographic characteristics (survey-based: number of children and ages, race/ethnicity, education, income, employment, marital status; electronic health record–based: maternal age)	N/A^b^		X			
	Baseline infant development	Mobility and behavior questions		X			
	Social support for PA	Modified family or friend support for participation in exercise scale [47,49]		X			
	COVID-19 stress and coping mechanisms	N/A		X			
	Health behaviors (smoking, alcohol, breastfeeding)	N/A		X	X	X	
**Adverse Event Reporting**							
	Injuries or illness related to exercise	N/A			X	X	

^a^Primary outcomes were depressive symptoms and device-based physical activity (PA) measured at the 3-month follow-up. Secondary outcomes were depressive symptoms and device-based PA at the 6-month follow-up and self-reported PA at the 3- and 6-month follow-ups.

^b^Not applicable.

**Table 2:** Electronic health record (EHR) identification of potentially eligible participants, study inclusion and exclusion eligibility criteria, recruitment and retention strategies and their modifications over the study period.

	Original Protocol—November 2020	Modification 1—January 2021	Modification 2—July 2021	Modification 3—February 2022
Identification of potentially eligible participants via the EHR	2-6 months post partum AND No current depression diagnosis AND Postpartum PHQ-9 score between 10 and 19 OR Postpartum PHQ-2 score ≥3	N/A^a^	2-6 months post partum AND No current depression diagnosis AND Postpartum PHQ-9^b^ score (10-19) OR Postpartum PHQ-2^c^ score ≥3 OR History of depression diagnosis or antidepressant medication use	N/A
Inclusion criteria Ascertained via eligibility screener	PHQ-8^d^ score between 10 and 19 Engages in <30 minutes of regular, moderate/vigorous intensity physical activity per week	PHQ-8 score between 10-19 Engages in <90 minutes per week of regular, moderate/vigorous intensity physical activity per week	Clarification of what is meant by moderate/vigorous intensity physical activity in eligibility screener	N/A
Exclusion Criteria Ascertained via eligibility screener	Not current Kaiser Permanente member <18 years of age Does not own a smartphone, computer, or TV with internet access Has a heart condition and a physician recommending medically supervised physical activity Has chest pain during physical activity or chest pain within the prior month Takes medication for hypertension or a heart condition Diagnosed with depression or received treatment for depression (eg, taken antidepressant medications or received psychotherapy) since giving birth Tendency to fall due to syncope or dizziness Has orthopedic problems that might be aggravated by physical activity Has exercise-induced asthma Is currently pregnant or is planning to become pregnant in the next 3 months Baby weighs outside 11-22 lbs Baby has a chronic illness/disorder that prevent them from being held or lifted up	N/A	N/A	N/A
Recruitment	Recruitment email followed up by recruitment phone call to provide opportunity to discuss the study DOB^e^ authentication by participants to enter survey Study hotline and email to respond to participant communication	N/A	Recruitment letter mailed first Recruitment email Recruitment phone calls made when recruiter time available Removed DOB authentication due to technical issues—recruiter confirms identity over phone Study website	N/A
Retention	Reminder phone calls to wear and send back activity monitor Financial incentive for completion of each assessment (US $70.00 in total) Recognizable logo and culturally competent and sensitive material	N/A	Information added to email for participants emphasizing the importance of contributions and survey completion for all participants, regardless of study group Study gift - Notepad with logo sent at 3 months Study newsletters with information on postpartum topics between study surveys Reminder emails/texts about follow-up surveys One reminder email/text 1 week after randomization to log in to MomZing in intervention group	Shorter, limited surveys, only including primary outcomes were available Additional reminders (text/email) were sent to intervention group for using MomZing

^a^N/A: not applicable.

^b^PHQ-9: Patient Health Questionnaire-9.

^c^PHQ-2: Patient Health Questionnaire-2.

^d^PHQ-8: Patient Health Questionnaire-8.

^e^DOB: Date of birth.

### EHR Identification of Potential Participants

The EHR was used as an efficient way to identify potentially eligible individuals to invite to participate. Recruitment began on November 19, 2020, and ended on September 2, 2022. Individuals who were 2-6 months post partum, did not have a current depression diagnosis and were at high risk for PPD (PHQ-9 score of 10-19 [35,36] or PHQ-2 score of ≥3 [39]) were identified in the EHR for potential recruitment. However, KPNC’s PPD screening rates were severely impacted by COVID-19’s effects on health care delivery. As a result, the number of potentially eligible participants identified was much lower than originally anticipated. Thus, in July 2021 we expanded our approach to include participants with a history of depression diagnosis or antidepressant medication use prior to the delivery date (Modification 2, see Table 2).

Individuals identified through the EHR were sent a recruitment email that included a link to the eligibility screener using email addresses registered in the EHR. Starting a week later, nonresponders were contacted by phone. Those meeting the eligibility criteria were invited to enroll.

### Clinician Identification of Potential Participants

As a secondary recruitment strategy, health system clinicians were encouraged to identify potential participants from their patient panel. Clinicians shared information about the trial with such patients and shared contact information with the study team. If the participant met the identification criteria, a study team member then reached out to the individual to screen for eligibility.

### Eligibility Criteria

Eligibility criteria are outlined in Table 2. Potential participants were screened for depressive symptoms using the validated Patient Health Questionnaire-8 (PHQ-8) [40]. The PHQ-8 is a validated instrument for assessing depressive symptoms similar to the PHQ-9 but does not assess suicidal ideation [50]. Scores between 10 and 19 (indicating high risk for PPD) were considered eligible. Low physical activity during postpartum was defined as not engaging in regular, moderate or vigorous intensity physical activity for 30 minutes or more per week. “High” physical activity (ie, 30 minutes or more per week) was the most common reason for ineligibility in the first few months of recruitment. On January 13, 2021 (Modification 1), the current low PA during postpartum criterion was modified to “not engaging in regular, moderate or vigorous intensity physical activity for 90 minutes or more per week” to match the American College of Sports Medicine’s definition of not participating in regular exercise [51].

### Recruitment

For individuals identified through the EHR, we sought approval to recruit from their obstetric provider (if a provider did not respond within 14 days, it was considered an approval to proceed) via email to contact the individual. Individuals were emailed recruitment materials with information about the trial, a link to the eligibility screener, and a link to the trial website [52]. Individuals were sent 1 recruitment email with a link to the eligibility screener requiring login using date of birth, followed by phone call recruitment if there was no response to the email. The recruitment protocol was modified (Modification 2, July 13, 2021) to first send a recruitment letter by postal mail followed by the original protocol. The date of birth authentication was also removed due to technical issues preventing participants from accessing the screener. Recruiters attempted to reach potential participants, (prioritizing PHQ-9 scores of 10-19) by phone at least once as recruiters’ time allowed. All recruitment efforts stopped once a patient reached 6 months post partum.

### Baseline Data Collection

Eligible participants completed Informed eConsent through REDCap (Research Electronic Data Capture) and were emailed a copy of the signed consent form. Next, the participant completed baseline surveys through REDCap. After baseline survey completion and mailing address confirmation, an accelerometer (Actigraph GT3X+) was mailed to the participant. Participants were asked to wear it for 24 hours for 7 consecutive days, complete a wear log for the activity monitor, and mail the activity monitor and log back. Once baseline surveys were completed and the activity monitor was returned, participants were then randomized into the intervention or usual care group.

### Randomization and Blinding

Participants were randomized using the minimization randomization technique as implemented via the QMinim software program [53,54], which was generated by the project manager [53,54]. Factors included in the block randomization included: parity (1 vs 2+), racial and ethnic category (Asian or Pacific Islander, Hispanic, non-Hispanic Black, non-Hispanic White, or Other), baseline PHQ-8 severity (scores of 10-14 and 15-19), and physical activity level prior to pregnancy (below vs at or above recommendations). Information on these factors was obtained from the eligibility screening and baseline trial questionnaires. The data analyst and investigators were blinded.

### Follow-Up Data Collection

**Figure 1:**
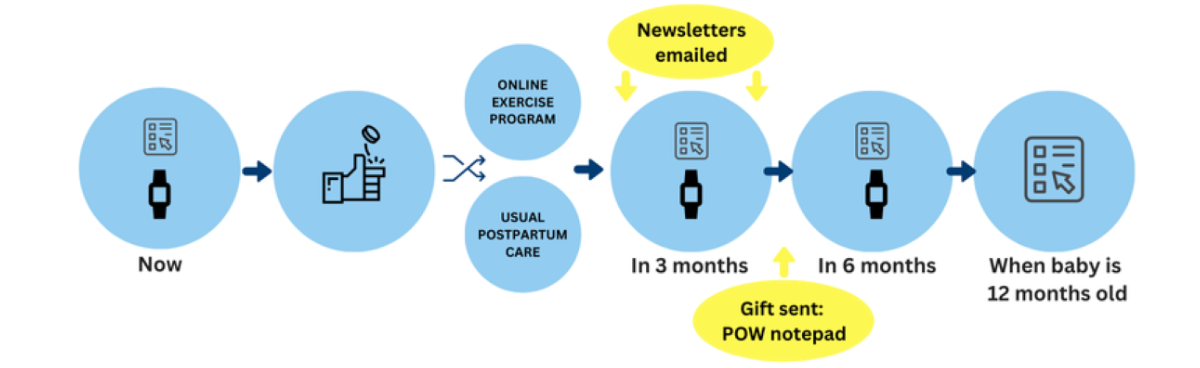
Final protocol. POW: POstpartum Wellness study.

Follow up data collection occurred at 3 and 6 months after randomization (Figure 1). An additional assessment occurred when the participant’s child was 12 months old; for some participants, this occurred simultaneously with the 6-month follow-up. At 3 and 6 months after randomization, participants were emailed links to web-based surveys via REDCap. After completion of the surveys at each timepoint, participants were sent an accelerometer (ActiGraph GT3X+), asked to wear it for 24 hours for 7 consecutive days, complete an activity monitor wear log, and mail back the activity monitor and wear log. When the participant’s child was 12 months old, participants were emailed the link to the web-based Ages and Stages Questionnaire (ASQ-3). Occasionally, the 6-month survey would fall around the time when the child was 12 months old. In these cases, the 6-month survey and ASQ-3 were sent together.

To increase retention and ascertainment of the primary outcomes (depression symptoms and PA) partway through the trial (Modification 3, February 2022), a limited survey was sent to participants nearing the end of the follow-up period and who had not completed their surveys.

### Retention Strategies

Strategies implemented mid-way through the trial to increase retention included newsletters, a gift, and text or email reminders (Table 2, Figure 1). Once implemented, participants received a newsletter halfway between randomization and the 3-month follow-up and then again halfway between the 3- and 6-month data collection points. The newsletters contained fun facts, tips for things such as bedtime and baby development, additional resources (unrelated to PA or depressive symptoms), and contact information for the study team. A week before each follow-up, participants would receive a notification text or email to expect their survey within a week. As a gift, participants received a notepad with the trial logo included with the 3-month follow-up activity monitor.

### Usual Care

Participants randomized to the usual care group received usual postpartum care for women at increased risk of depression, which typically is a brief discussion about their depression symptoms with their obstetric provider.

### Intervention

**Figure 2:**
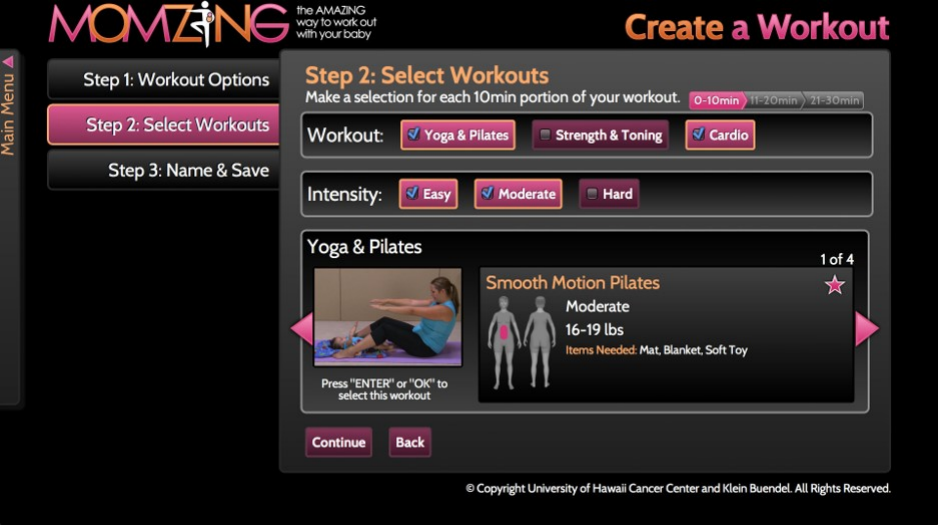
Screenshot of MomZing program.

Participants randomized to the intervention group received usual care plus access to MomZing (Figure 2) [55], a web-based library of tailored exercise videos [56] that was developed based on postpartum individuals’ preferences for exercise videos that (1) guided them on how to exercise safely with their baby based on the infant’s weight and developmental stage; (2) did not require exercise equipment or a substantial time commitment per video (eg, maximum time per video of 10 minutes); (3) provided different types of physical activities (yoga, strengthening, cardio) and intensity levels (light, moderate, hard); and finally, (4) featured women in the exercise demonstrations who were “real” postpartum individuals (not fitness instructors) exercising with their own infant. Users could either select individual videos, combine up to 3 videos to create a longer workout, or choose a “Ready Made” workout lasting 10, 20, or 30 minutes. The website also included an activity tracker that logged exercise videos watched and allowed users to input outside workouts to track daily and weekly total physical activity. Participants randomized to the intervention group were provided individual login information for the website.

Adherence and engagement with the intervention were assessed using website analytics to track logins and videos watched. The 3-month follow-up survey for participants in the intervention group included additional questions on intervention satisfaction.

To increase adherence to the intervention, after Modification 2, participants randomized to the intervention group received 1 text or email reminder (depending on participants’ preference) 1 week after randomization to log into the MomZing website. After Modification 3, additional text reminders were sent to participants in the intervention group between randomization and 3-month follow-up, reminding them to log in to the MomZing website in an effort to increase use of the intervention. Participants could receive up to 5 additional reminder texts at the discretion of research staff.

### Outcomes

#### Primary and Secondary Outcomes

Primary outcomes were depressive symptoms and device-based PA measured at the 3-month follow-up. Secondary outcomes were depressive symptoms and device-based PA at the 6-month follow-up and self-reported PA at the 3- and 6-month follow-ups.

#### Depressive Symptoms

Depressive symptoms were measured at baseline (eligibility screening), 3-month, and 6-month follow-up using the PHQ-8 [40]. The PHQ-8 has been validated in many studies as an instrument for screening for depression with high sensitivity (>88%) and specificity (>88%) in obstetric patients. The PHQ-8 is also a valid tool to establish depression severity and outcome [40]. The 8-question screener scores range from 0 to 24. A score of 1-4 suggests minimal depression; 5-9, mild depression; 10-14, moderate depression; 15-19, moderately severe depression; and 20-24, severe depression.

#### Device-Measured Moderate or Vigorous Intensity PA

Device-measured moderate or vigorous intensity PA (dm-MVPA) was measured using accelerometry at baseline, 3-month, and 6-month follow-up. The Choi algorithm [57-59], Tracy algorithm [60], and the Hibbing two-regression model, which was developed for wrist-worn accelerometer data [61], were used to identify wear time, bedrest, and PA intensity, respectively. Average moderate or vigorous intensity PA duration was calculated across valid days.

#### Self-Reported Moderate or Vigorous PA

Self-reported moderate or vigorous intensity PA (sr-MVPA) was assessed using the sports or exercise domain of the Pregnancy Physical Activity Questionnaire [41], a valid and reliable instrument developed for perinatal populations.

#### Additional Outcomes

##### Sleep

The 19-item Pittsburgh Sleep Quality Index (PSQI) [44] was used to measure sleep during the past month. A global score, ranging from 0 to 21, is calculated using 7 components of sleep. Higher scores indicate poorer sleep quality.

##### Anxiety

The Generalized Anxiety Disorder Scale (GAD-7) has been validated in prenatal [62] and racially diverse populations [63,64] and was used to measure anxiety symptoms. Scores range from 0 to 21 and scores of 10-21 were categorized as clinically significant anxiety symptoms [43].

##### Perceived Stress

The Perceived Stress Scale (PSS-10) [42] is the most widely used psychological instrument for measuring the perception of stress and has been validated in diverse populations and in perinatal women [65]. Scores range from 0 to 40 with 14 or greater signifying moderate-to-severe perceived stress.

##### Parent-Infant Bonding

The Mother-to-Infant Bonding Scale (MIBS) [45] is an 8-item questionnaire designed to assess feelings of the birthing parent toward their baby. The MIBS has demonstrated acceptability and has good internal reliability. Scores range from 0 to 24, with lower scores indicating better parent-infant bonding.

##### Infant Neurodevelopment

Infant neurodevelopment at 12 months was assessed using the validated ASQ-3 [46], a high-quality tool to screen for developmental delays in children [46,66]. The ASQ-3 screens for delays in child development in 5 domains: communication, gross motor, fine motor, problem-solving, and personal adaptive skills. Scores will be calculated based on the ASQ-3 scoring guide with scores above the cut-off point indicating typical development categorized as “on schedule” and scores in the zones indicating the need for monitoring or the need for further assessment categorized as “not on schedule.”

### Sample Size

Original sample size calculations were based on a planned sample size of 100 participants per group. With our achieved sample size of 99 participants (50 in the intervention group, 49 in the control group), 80% power, and 5% type I error rate, the minimum detectable difference in mean depressive symptom scores and accelerometer-measured duration of moderate or vigorous intensity PA was 0.57 standard deviation units, which is considered a “medium” effect size [67].

### Data Analysis

Demographic characteristics for those invited to participate in the trial and those enrolled and randomized are presented in Table 3 and baseline characteristics of the intervention and control groups are presented in Table 4. Intention-to-treat will be used for primary data analysis. We will use linear regression models for estimation of mean differences in outcomes between the intervention and control group, adjusting for all variables included in the randomization scheme.

**Table 3:** Participant characteristics for postpartum individuals invited and those randomized.

Characteristic		Invited (n=12,269)	Randomized (n=99)
Age (years), mean (SD)		31.5 (5.4)	32.1 (4.8)
Months post partum at recruitment letter sent, mean (SD)		2.36 (0.66)	2.43 (0.78)
**Race/ethnicity, n (%)**			
	Asian or Pacific Islander	2141 (17)	10 (10)
	Hispanic	3615 (29)	31 (31)
	Multiracial	416 (3.4)	5 (5.1)
	Native American	55 (0.4)	1 (1.0)
	Non-Hispanic Black	1075 (8.8)	7 (7.1)
	Non-Hispanic White	4738 (39)	44 (44)
	Other or unknown	229 (1.9)	1 (1.0)
**Parity, n (%)**			
	0	4690 (38)	40 (40)
	1+	6917 (56)	55 (56)
	Unknown	662 (5.4)	4 (4.0)
**Marital Status, n (%)**			
	Married, registered domestic partner, or common law	7477 (61)	65 (66)
	Separated or divorced	194 (1.6)	1 (1.0)
	Single or never married	4223 (34)	33 (33)
	Widowed or Other	72 (0.6)	0 (0)
	Unknown	303 (2.5)	0 (0)
Medicaid insurance, n (%)		1842 (16)	10 (10)
PHQ-2^a^ Score, mean (SD)		3.72 (0.91)	3.65 (0.74)
PHQ-9^b^ Score, mean (SD)		12.71 (2.49)	12.18 (2.30)

^a^PHQ-2: Patient Health Questionnaire-2.

^b^PHQ-9: Patient Health Questionnaire-9.

**Table 4:** Baseline participant sociodemographic characteristics by randomization arm.

Characteristic			Control (n=49)		Intervention (n=50)
Baseline PHQ-8^a^ score^b^, mean (SD)			12.6 (2.2)		12.6 (2.2)
Age at randomization (years), mean (SD)			32.7 (4.5)		31.9 (5.1)
Months post partum at randomization, mean (SD)			4.0 (1.0)		4.1 (1.2)
**Race and ethnicity^b^, n (%)**					
	Asian or Pacific Islander	4 (8.2)		6 (12)	
	Hispanic	12 (24)		10 (20)	
	Non-Hispanic Black	3 (6.1)		3 (6.0)	
	Non-Hispanic White	18 (37)		19 (38)	
	Other^c^	12 (24)		12 (24)	
**Highest level of education, n (%)**					
	High School or less	8 (16)		11 (22)	
	College	29 (59)		31 (62)	
	Graduate School	12 (24)		7 (14)	
	Unknown	0 (0)		1 (2.0)	
**Annual household income (US $), n (%)**					
	Less than 65,000 per year	13 (27)		13 (26)	
	65,000 to 99,999 per year	9 (18)		14 (28)	
	100,000 and greater per year	23 (47)		17 (34)	
	Unknown	4 (8.2)		6 (12)	
**Employment status, n (%)**					
	Currently working	15 (31)		11 (22)	
	Not currently working	34 (69)		39 (78)	
**Marital status, n (%)**					
	Married, civil union, or living with a partner	44 (90)		41 (82)	
	Single or divorced	5 (10)		9 (18)	
**Number of children at home^b^, n (%)**					
	>1	28 (57)		30 (60)	
	1	21 (43)		20 (40)	
**Prepregnancy activity level^b^, n (%)**					
	At or above recommendations^d^	18 (37)		21 (42)	
	Below recommendations	31 (63)		29 (58)	

^a^PHQ-8: Patient Health Questionnaire-8.

^b^Variable included in randomization schema.

^c^Other includes Multiracial, Native American, and unknown.

^d^Moderate or higher intensity physical activity ≥5 times per week for ≥30 minutes a time.

### Ethical Considerations

The study protocol was approved by the Kaiser Permanente Northern California Institutional Review Board (#1548855). All participants provided documented informed consent. It was explained to them that they could withdraw from the project at any time for any reason without any repercussions. Confidentiality of participation was maintained, with data access exclusively limited to the research team members. Participants’ personal identity was not revealed during data collection, analysis, presentations, and publications. Participants received gift cards after completing each assessment: US $20 after the baseline and 3-month assessments and US $30 after the 6-month assessment (including the 12-month infant ASQ screener), for a total of US $70.

## Results

**Figure 3:**
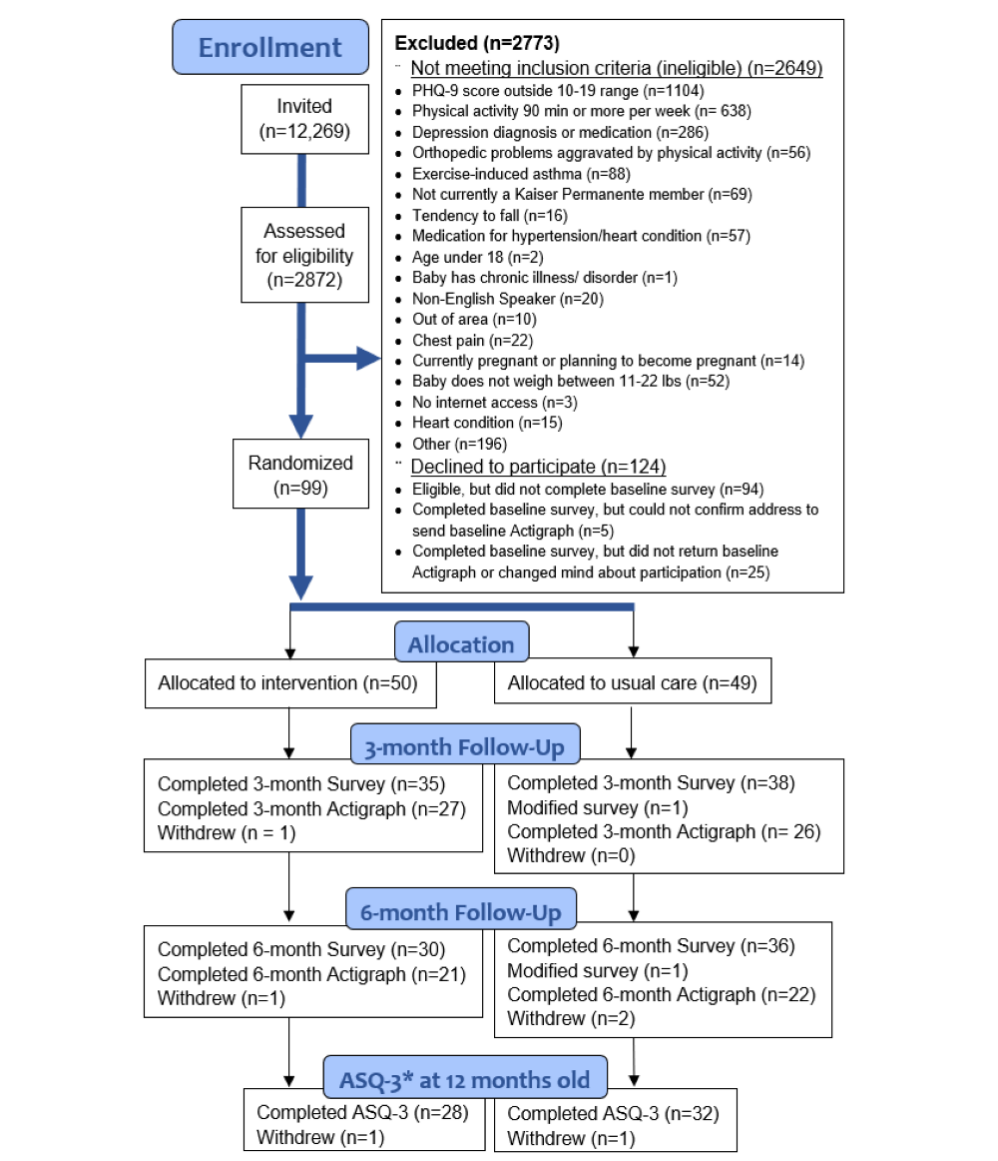
CONSORT (Consolidated Standards of Reporting Trials) flow diagram. ASQ-3: Ages and Stages Questionnaire, Third Edition.

The study was funded in January 2020. During 22 months of recruitment (November 2020-September 2022), 12,269 postpartum KPNC members were invited to participate in this trial (Table 3). The recruited sample was representative of all participants invited to participate in the trial with regard to several sociodemographic characteristics and depressive symptoms captured in the EHR with the exception of race and ethnicity (Table 3). A smaller percentage of Asian or Pacific Islander individuals were randomized compared to those invited (10% vs 17%) and a larger percentage of Non-Hispanic White participants were recruited compared to invited (44% vs 39%). Of the 2872 postpartum individuals assessed for eligibility, 2773 were not eligible, 124 declined to participate, and 99 were randomized (50 to intervention and 49 to usual care; Figure 3).

### Baseline Characteristics

The randomized sample included 55 (56%) participants from racial and ethnic backgrounds other than non-Hispanic White (Table 4). Participants were 4.0 (SD 1.0) months post partum on average at baseline with moderately severe depressive symptoms (mean PHQ-8 score 12.6, SD 2.2). The intervention and usual care groups were similar regarding sociodemographic characteristics, months post partum, baseline depressive symptoms, number of children at home, and prepregnancy PA level.

### Retention

Follow-up data collection ended in April 2023. At the 3-month follow-up, 73 out of 99 (74%) participants (35/50, 70% in the intervention group and 38/49, 78% in the usual care group) completed questionnaires; 53 out of 99 (54%) returned the accelerometer after wearing it for 7 days (27/50, 54% in the intervention group and 26/49, 53% in the usual care group). At the 6-month follow-up, 66 out of 99 (67%) participants (30/50, 60% in the intervention group and 36/49, 73% in the usual care group) completed questionnaires and 43 out of 99 (43%) returned the accelerometer after wearing it for 7 days (21/50, 42% in the intervention group and 22/49, 45% in the usual care group). A total of 60 out of 99 (61%) participants completed the ASQ-3 when their child was 12 months old (28/50, 56% in intervention group and 32/49, 65% in the usual care group).

### Impact of Modifications on Recruitment and Retention

Modification 1 reduced the number of participants ineligible due to “high” PA (21/59, 36% before vs 689/2813, 25% after); however, the overall eligibility rate only changed slightly (7/59, 12% before vs 216/2813, 8% after) as many participants remained ineligible due to PHQ-8 scores outside of the eligible range (15/59, 25% before vs 1089/2813, 39% after).

Modification 2 increased the monthly recruitment rate (4.2 participants per month before vs 6.2 participants per month after) and the proportion of participants recruited by email (3/30, 10% before vs 39/69, 56% after), added postal recruitment (25 participants) and decreased the proportion of participants recruited by phone (27/39, 90% vs 5/69, 7%). Retention rates decreased for the 3-month follow-up survey after Modification 2 (20/21, 95% before vs 53/78, 68% after). Retention increased for the 6-month follow-up survey after Modification 2 (2/4, 50% before vs 64/93, 69% after).

Modification 3 increased the proportion of participants in the intervention group that logged into the MomZing website (11/18, 61% before vs 29/32, 91% after) and decreased the mean time between randomization and first login (22 days before vs 8 days after). Analysis of the effectiveness of the MomZing intervention has been completed, and a manuscript with these findings is currently under review.

## Discussion

### Background

Interventions to prevent PPD that are feasible in the clinical setting and do not involve intensive health care system resources are urgently needed. The POW trial was designed to evaluate the effectiveness of an eHealth PA intervention for improving depressive symptoms and increasing PA among postpartum individuals at high risk for PPD. In addition to possible effects of the intervention on depressive symptoms and PA, the POW trial data can also be used to evaluate the effect of the eHealth PA intervention on postpartum sleep, perceived stress, anxiety symptoms, parent-infant bonding, and infant development. Given that the intervention tested here does not require intense health system resources, it has the potential for being adopted into clinical practice if found effective in decreasing PPD symptoms and increasing PA.

### Recruitment and Retention Strategies

Over the course of recruitment, we modified our protocol at several points in an attempt to increase recruitment, retention, and adherence to the intervention. Successful strategies included adding a recruitment letter sent via postal mail before sending the same letter via email and removing authentication to access surveys, which increased monthly recruitment rate and email recruitment and decreased staff time spent on phone recruitment, and adding reminders to log in to the MomZing website in the intervention group, which increased initial login rates and decreased time to first login from randomization. However, our modifications to the low PA eligibility criterion did not have the intended impact on eligibility rates. Although the ineligibility rate due to high physical activity decreased, most participants remained ineligible due to PHQ-8 scores outside of the eligible range. After modifications addressing retention (adding information emphasizing importance of survey completion for all participants, study gift, newsletters, and reminders about follow-up surveys) were implemented, retention rates did not improve for the 3-month follow-up and increased for the 6-month follow-up. Recent studies of retention strategies for RCTs suggest that study gifts and electronic or text reminders are effective retention strategies [68,69]. However, these may be less effective during postpartum in those with high risk of PPD, especially during a global pandemic.

Our retention rates ranged from 60% (30/50) to 78% (38/49) for questionnaire completion and 42% (21/50) to 54% (27/50) for accelerometer return, with retention rates decreasing from the 3-month to the 6-month follow-up. Compared to a previous RCT of a home-based eHealth PA intervention conducted among postpartum individuals at high risk of PPD [70], our retention rates are lower for questionnaire completion (70% (35/50) to 78% (38/49) versus 80%-97%) and higher for accelerometer data (53% (26/49) to 54% (27/50) versus 27%-47%) at 3-month follow-up. In an RCT of a team-based eHealth PA intervention in the general postpartum population, retention rates similarly decreased from 6 weeks to 6 months follow-up [71]. In our study, both the 3-month and 6-month follow-up retention rates for questionnaire completion were higher in the usual care arm than the intervention arm, while retention rates for accelerometer return were similar in both arms. A similar pattern was observed previously in an RCT of a mobile app targeting PA in postpartum [72]; however, other RCTs have observed the opposite pattern. In a previous RCT of a home-based eHealth PA intervention conducted among postpartum individuals at high risk of PPD [70] and an RCT of a team-based eHealth PA intervention in the general postpartum population [71], retention rates were higher in the intervention group than in the control group.

Additional strategies to improve recruitment, retention, and adherence outcomes that could be used in future studies include consultation with diverse stakeholders during the design study phase [72]; larger financial incentives [73]; trial orientation sessions [74]; and methodological infographics that convey the scientific importance of high, nondifferential trial retention [75].

### Strengths

Strengths of our trial include recruitment of individuals within an integrated health care delivery system with universal PPD screening, allowing for efficient identification of patients within the KPNC health care system who meet the criteria for high risk of PPD to participate in this trial; recruitment of a representative sample of postpartum individuals at high risk of PPD; use of an eHealth PA intervention for postpartum individuals that was developed with key stakeholder input, resulting in a tailored intervention for postpartum individuals; inclusion of several important potential confounders in our randomization scheme to ensure that randomized groups are balanced; and assessment of multiple behaviors, mental health outcomes, and infant development at multiple timepoints, which will allow for assessment of longitudinal effects of the intervention across postpartum. Finally, 80% of participants randomized to the intervention group logged into the MomZing website; however, additional metrics of engagement will be considered to determine the impact of adherence and engagement with the intervention on results. Results will be published in the peer-reviewed scientific literature.

### Limitations

Although the research team worked diligently to monitor trial implementation, remain flexible [76], include a diverse and sensitive research staff [76], and introduce new strategies in an effort to improve recruitment, retention, and adherence outcomes throughout the trial, we acknowledge a few limitations with our trial. The challenges we encountered are common in clinical trials [77]. While over 50% of our study sample was from a racial and ethnic minority group, the sample was not completely reflective of the racial and ethnic demographics of our target population and we had difficulty meeting our original recruitment goal. We noted differential follow-up rates by randomization arm, with predominately higher rates in the usual care arm which may result in biased estimates if there are differences between participants who completed the trial and those who were lost to follow-up. We will carefully consider loss-to-follow-up in our analyses and apply appropriate statistical methods to mitigate some of these concerns.

### Conclusions

In summary, the POW trial is designed to expand our understanding of the effectiveness of an eHealth PA intervention to reduce PPD symptoms and increase PA. The rigorous evaluation of this technology-based intervention that requires few resources from the health care system is designed to produce generalizable results to inform clinical practice. Throughout the study, we used flexible solutions to address challenges related to recruitment and retention, which are common in RCTs. Successful strategies included multiple modes of contact for recruitment (postal mailing, email, and phone calls) and reminders in the intervention group to log in to the intervention website. Differential follow-up rates will be addressed using appropriate statistical techniques to mitigate potential bias. Future studies of eHealth PA interventions in postpartum populations at high risk for PPD should monitor recruitment and retention frequently and be flexible in implementing strategies to increase recruitment, retention, and adherence to interventions.

## Abbreviations

ASQ-3: Ages and Stages Questionnaire
CONSORT: Consolidated Standards of Reporting Trials
dm-MVPA: device-measured moderate/vigorous intensity PA
EHR: electronic health record
GAD-7: Generalized Anxiety Disorder Scale
KPNC: Kaiser Permanente Northern California
MIBS: Mother-to-Infant Bonding Scale
PA: physical activity
PHQ-2: Patient Health Questionnaire-2
PHQ-8: Patient Health Questionnaire-8
PHQ-9: Patient Health Questionnaire-9
POW: POstpartum Wellness study
PPD: postpartum depression
PSQI: Pittsburgh Sleep Quality Index
PSS-10: Perceived Stress Scale
RCT: randomized controlled trial
REDCap: Research Electronic Data Capture
sr-MVPA: self-reported moderate or vigorous intensity physical activity

## Data Availability

The data sets generated during and analyzed during this study are available from the corresponding author on reasonable request.
